# Supplementary material for: The Construction and Exploration of a Comprehensive MicroRNA Centered Regulatory Network in Foxtail Millet (Setaria italica L.)
Source: Front Plant Sci. 2022 May 6;13:848474. doi: 10.3389/fpls.2022.848474 (PMC9121102; doi:10.3389/fpls.2022.848474)
Supplement: Supplementary file 3 [file Data_Sheet_3.zip › Sit-miR528_Seita.9G009600.1_69_TPlot.pdf]

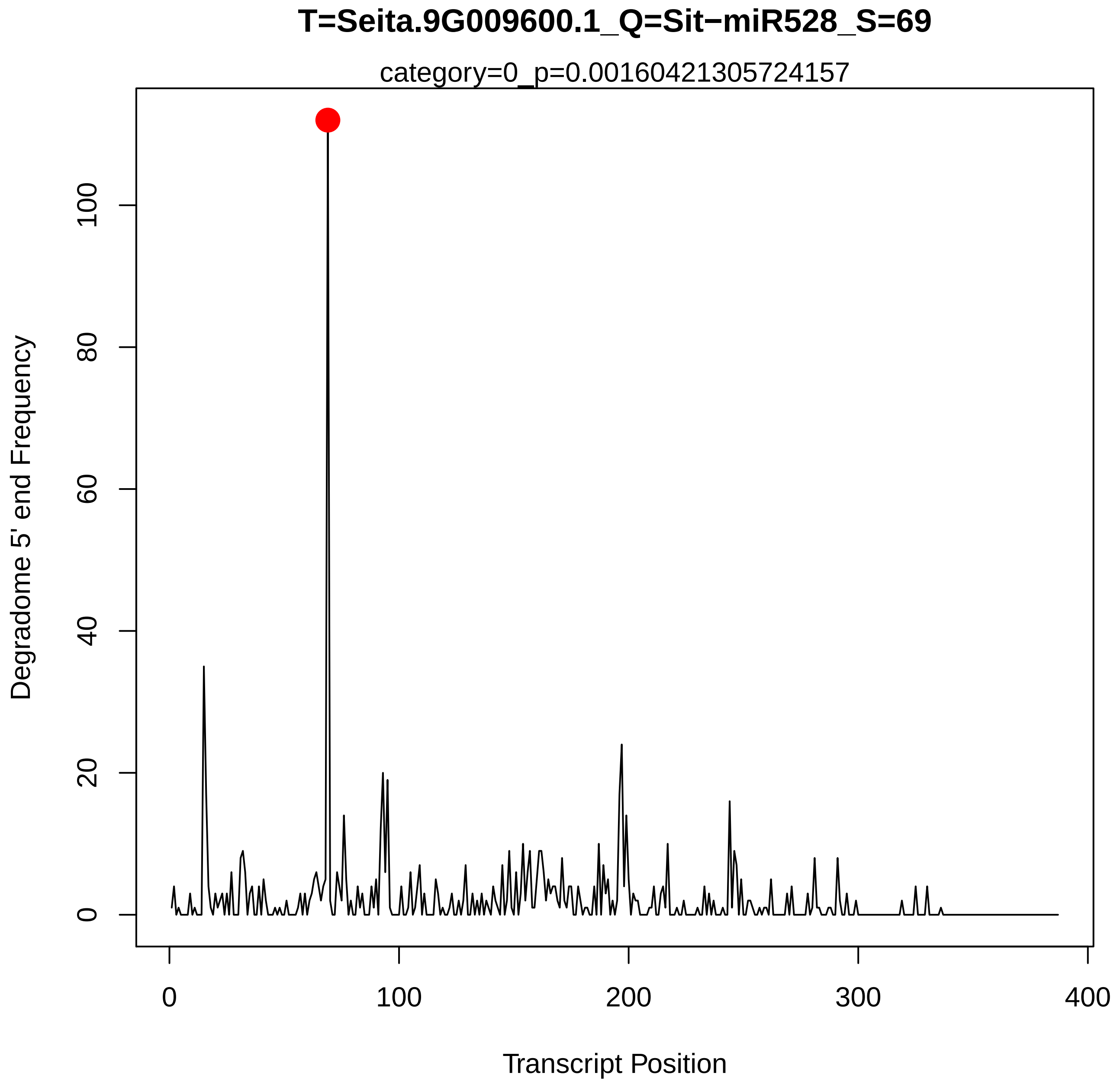
T=Seita.9G009600.1_Q=Sit−miR528_S=69
category=0_p=0.00160421305724157
Degradome 5' end Frequency
0
20
40
60
80
100
0
100
200
300
400
Transcript Position
